# Sex-dependent modulation of CGRPergic neurovascular activity by 5-CT in rats

**DOI:** 10.3389/fphar.2026.1766406

**Published:** 2026-04-28

**Authors:** Anaïs Clara Terol-Úbeda, Asunción Morán, Mónica García-Domingo, José Ángel García-Pedraza

**Affiliations:** 1 Laboratorio de Farmacología, Departamento de Fisiología y Farmacología, Facultad de Farmacia, Universidad de Salamanca, Salamanca, Spain; 2 Instituto de Investigación Biomédica de Salamanca (IBSAL), Salamanca, Spain

**Keywords:** 5-CT, 5-HT_1F_ receptor, 5-HT_7_ receptor, CGRP, migraine, sex differences, vasodepressor sensory outflow

## Abstract

**Background and purpose:**

Serotonin modulates vascular tone both directly and indirectly through autonomic and sensory nerves innervating blood vessels. Perivascular sensory nerves release calcitonin gene-related peptide (CGRP), a potent vasodilator strongly implicated in migraine pathophysiology. In male rats, the serotonergic system inhibits CGRPergic vasodepressor responses via 5-HT_1B/1F_ and 5-HT_7_ receptors. Since both serotonergic and CGRPergic pathways exhibit marked sex differences, the present study investigated the 5-HT receptor (sub)types involved in the 5-carboxamydotryptamine (5−CT, 5-HT_1/5/7_ receptor agonist) modulation of vascular CGRPergic neurotransmission in rats, focusing on sex-dependent differences.

**Methods:**

Male and female Wistar rats (14–16 weeks old) were pithed and pretreated with an i.v. continuous infusion of hexamethonium and methoxamine, followed by administration of 5-HT-related drugs. Mean blood pressure (MBP) and heart rate (HR) were continuously recorded throughout the experiments. Vasodepressor CGRPergic responses were elicited by electrical stimulation of the sensory outflow (0.1–5 Hz) or i.v. α-CGRP (0.1–1 μg/kg).

**Results:**

Basal MBP and HR were lower in females than in males, whereas the methoxamine-induced increase in MBP was greater in females. The electrically evoked vasodepressor responses, as well as their inhibition by 5−CT, were similar in both sexes. In males, the inhibitory effect of 5−CT was reproduced by 5-HT_1B_, 5-HT_1F_, and 5-HT_7_ receptor agonists (CP-93,129, LY344864, and AS-19, respectively) and persisted in the presence of the 5-HT_5A_ receptor antagonist SB699551. In contrast, in females, 5-CT-induced inhibition was mimicked by 5-HT_1F_ and 5-HT_7_ receptor agonists and was not affected by administration of SB699551. None of the other 5-HT receptor agonists (5-HT_1A/1B/1D_) modified the CGRPergic vasodilator responses in females. Only AS-19 reduced the vasodepressor responses elicited by exogenous α-CGRP in females.

**Conclusion:**

5−CT inhibits perivascular sensory CGRPergic neurotransmission in both male and female rats. Unlike males, where the 5−CT effect is mediated by prejunctional 5-HT_1B/1F/7_ receptors, in females, this inhibitory effect is mediated by prejunctional 5-HT_1F_ and pre and/or postjunctional 5-HT_7_ receptors. These findings provide novel insights into sex-specific serotonergic modulation of neurovascular function.

## Graphical Abstract

**Figure:**
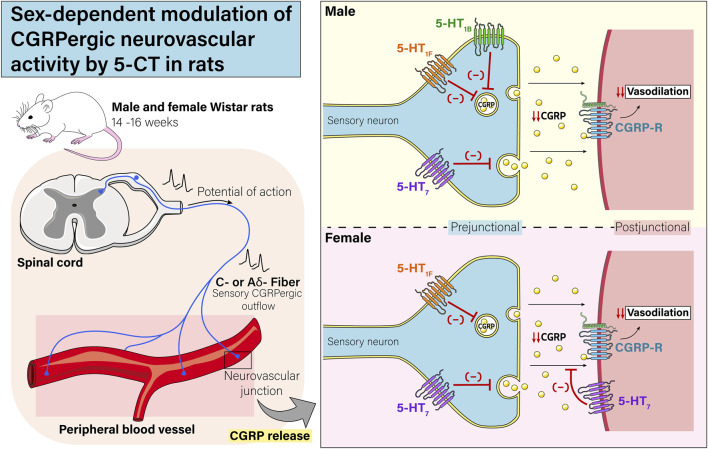

## 1 Introduction

In addition to its prominent role in the central nervous system (CNS), serotonin (5-hydroxytryptamine, 5-HT) participates in the regulation of gastrointestinal motility, endocrine function and cardiovascular homeostasis (Nichols and Nichols, 2008), in which 5-HT exerts multifaceted and even biphasic effects on vascular function (vasoconstriction/vasodilation). This complexity is due to (i) the ability of 5-HT to act at multiple levels–including the heart, vascular endothelial and smooth muscle cells, and peripheral autonomic and sensory nervous systems; and (ii) the involvement of its multiple receptor types and subtypes (González-Hernández et al., 2023), since seven major classes of 5-HT receptors have been identified, namely from 5-HT_1_ to 5-HT_7_ receptors, with a total of 14 receptor subtypes (Barnes et al., 2021; Feng et al., 2025; Zhang et al., 2025).

Perivascular sensory nerves, mainly C and Aδ fibers, play an important role in the regulation of vascular tone and the maintenance of blood pressure (BP) (Cuesta et al., 2014; González-Hernández et al., 2016; Thakore and Brain, 2017). These nerves release potent vasodilatory peptides, with substance P and calcitonin gene-related peptide (CGRP) as the principal mediators (González-Hernández et al., 2016; Thakore and Brain, 2017). CGRP receptors are expressed throughout the central and peripheral nervous systems, including the trigeminovascular pathways, where their activation is strongly linked to migraine pathophysiology (Hay and Walker, 2017). In male pithed rats, electrical stimulation of perivascular sensory nerves induces vasodilation via CGRP release (Lozano-Cuenca et al., 2009; González-Hernandez et al., 2010; 2011; Cuesta et al., 2014; Miguel-Martínez et al., 2023). Interestingly, these non-adrenergic non-cholinergic (NANC) responses can be inhibited prejunctionally by activation of 5-HT_1B/1F_ (González-Hernandez et al., 2010; 2011) and 5-HT_7_ (Cuesta et al., 2014) receptors.

Migraine, a highly prevalent neurovascular disorder affecting approximately 15% of the global population (Ferrari et al., 2022), is the main contributor to headache disorders, which rank as the third leading cause of years lived with disability globally (GBD, 2021 Headache Collaborators, 2025). During a migraine attack, increased CGRP release from trigeminal sensory neurons promotes vasodilation, neurogenic inflammation and nociceptive transmission (Bonura et al., 2023; Pleş et al., 2023). Accordingly, pharmacological modulation of CGRP signaling represents a major therapeutic strategy in migraine management (Lewter et al., 2025).

Sex-related differences have been reported in several neurovascular (Maddahi et al., 2023; de Vries Lentsch et al., 2021) and autonomic pathways (Dearing et al., 2022; Terol-Úbeda et al., 2025b). CGRP-mediated vasodilation appears to be influenced by female sex hormones. Plasma levels of CGRP are higher in women than in men, and cyclic fluctuations of ovarian hormones modulate CGRP both in peripheral and CNS (Favoni et al., 2019). Indeed, starting during puberty, migraine occurs in women three to four times more often than in men, and this bias decreases after menopause (Russo and Hay, 2023). Accordingly, the Global Burden of Disease Study (2019) ranked migraine as the leading cause of disability among women in reproductive age (15–49 years) worldwide (Steiner et al., 2020; Krause et al., 2021).

Our group has recently shown that serotonergic contribution to cardiovascular homeostasis is also sex dependent, as demonstrated in its modulation of vascular sympathetic neurotransmission (Terol-Úbeda et al., 2025a; Terol-Úbeda et al., 2025b). Considering that 5-HT_1_ and 5-HT_7_ receptor families play a predominant role in the serotonergic inhibition of vasodepressor sensory CGRPergic outflow in male rats, the present study aimed to investigate the 5-HT receptor (sub)types involved in the 5-carboxamydotryptamine (5−CT, 5-HT_1/5/7_ receptor agonist)-induced modulation of vascular CGRPergic neurotransmission in rats, with particular emphasis on sex-dependent differences.

## 2 Materials and methods

### 2.1 Compounds

Each drug and its respective supplier used in the study were: sodium pentobarbital (Dolethal®; Vetoquinol; Madrid, Spain); sodium heparin (Rovi; Madrid, Spain); atropine sulphate (Scharlab; Barcelona, Spain); d-tubocurarine hydrochloride, hexamethonium bromide and methoxamine hydrochloride (Merck Life Science S.L.U.; Madrid, Spain); 5−CT maleate, 8-OH-DPAT, AS-19, α-CGRP, CP-93,129 dihydrochloride, GR127935 hydrochloride, LY344864 hydrochloride, SB258719 hydrochloride, SB699551 and sumatriptan succinate (Tocris Bioscience; Bristol, UK).

All these compounds were dissolved in saline at the experimentation time, except AS-19 (dissolved in ethanol 5% (EtOH)).

### 2.2 Animal preparation and study design

Male (n = 40) and female (n = 75) Wistar rats (14–16 weeks old, 250 ± 25 g) were obtained and housed in the animal facility of the University of Salamanca until the time of experimentation. Animals were co-housed in groups of up to five per cage under controlled environmental conditions (22 °C ± 2 °C, 50% humidity, 12-hour light/dark cycle) and had unrestricted access to food and water.

Rats were anaesthetised with pentobarbital (60 mg/kg) via intraperitoneal injection. Once withdrawal reflexes were no longer present, a cannula was placed in the trachea and the animals were pithed by inserting a stainless-steel rod through the right orbital sinus and foramen magnum and down into the spinal cord, as previously established (Cuesta et al., 2014; Terol-Úbeda et al., 2025a; Terol-Úbeda et al., 2025b). Then, rats were artificially ventilated with room air (50 strokes/min, 1 mL air/100 g) and catheters were placed in (i) jugular and femoral veins, for the continuous perfusion of agonists (methoxamine followed by 5-HT receptor agonists) and i.v. administration of 5-HT receptors antagonists; and (ii) the left carotid artery, coupled to a pressure transducer and connected to an e-corder 410 amplifier, for recording mean BP (MBP; mmHg) and heart rate (HR; beats/min, bpm) using Chart^TM^ (v5.5.11; eDAQ) and LabChart^TM^ (v7.2; ADInstruments) software. Coagulation of the blood was avoided by the i.v. injection of heparin (1000 IU/kg) and cholinergic effects were blocked with atropine (1 mg/kg i.v.).

**FIGURE 1:**
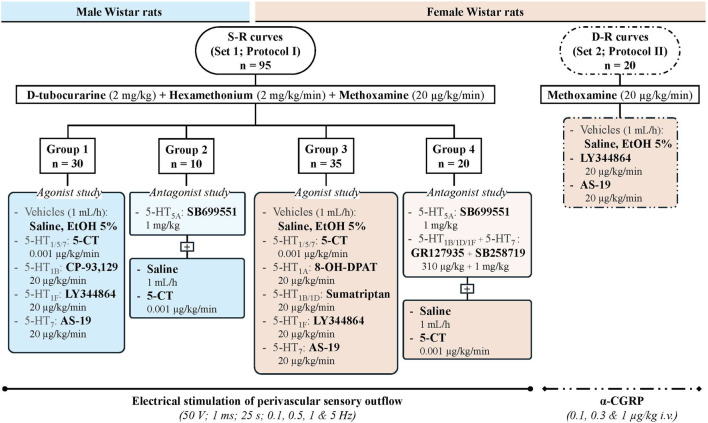
Study design showing the number of animals used in the two main sets and their subdivisions (n = 5 each). Vasodepressor responses were obtained either by electrical stimulation of the perivascular sensory outflow (set 1; groups 1–2: male rats; groups 3–4: female rats) or i.v. bolus administration of exogenous α-CGRP (set 2: female rats). D-R curve: dose-response curve; EtOH: ethanol; S-R curve: (electrical) stimulus-response curve.

At this point, rats were divided into two main sets (Figure 1), in order to study the effect produced by the continuous infusion of 5-HT agonists on the vasodepressor responses induced by (i) electrical stimulation of perivascular sensory outflow (set 1; n = 95) or (ii) i.v. administration of exogenous α-CGRP (set 2; n = 20). The vasodepressor stimulus-response curves (S-R curves) and dose-response curves (D-R curves) induced by electrical stimulation and exogenous α-CGRP, respectively, were completed in about 50 min with no significant changes in baseline MBP and HR. As previously reported (Cuesta et al., 2014; Miguel-Martínez et al., 2023), only one S-R curve or D-R curve was performed per animal as tachyphylaxis of the CGRPergic vasodepressor responses was observed when eliciting a second S-R or D-R curve, and the time lapse between the different stimulation frequencies (0.1, 0.5, 1 and 5 Hz) or α-CGRP doses (0.1, 0.3 and 1 μg/kg) was approximately 5 min, when MBP had returned to baseline values after vasodepressor responses.

### 2.3 Experimental protocols

Once a stable haemodynamic condition had been maintained for at least 10 min, baseline MBP and HR values were recorded, and the animals were randomly assigned to either Protocol I or Protocol II (Figure 1).

#### 2.3.1 Protocol I. Electrical stimulation of the perivascular sensory outflow

Prior to electrical stimulation, the first set of animals received the following i.v. treatments: (i) a bolus administration of d-tubocurarine (2 mg/kg) to prevent skeletal muscle contraction during electrical stimulation of the spinal cord; (ii) 10 min later, a continuous perfusion of hexamethonium (2 mg/kg/min) to block vasopressor responses induced by stimulation of the preganglionic sympathetic vasopressor outflow; and (iii) after an additional 15 min, a continuous infusion of methoxamine (20 μg/kg/min) to increase MBP (Villalon et al., 2008; Cuesta et al., 2014; Miguel-Martínez et al., 2023). After 25 min, baseline values of MBP and HR were assessed. Then, animals were distributed into four experimental groups by sex (groups 1–2: males; groups 3–4: females; Figure 1).

The first group (Group 1; male rats; n = 30) was divided into six subgroups (n = 5 each) that received the following continuous i.v. perfusions: (a) saline, (b) EtOH 5% (vehicles; 1 mL/h) or selective 5-HT receptor (sub)type agonists: (c) 5−CT (5-HT_1/5/7_; 0.001 μg/kg/min), (d) CP-93,129 (5-HT_1B_; 20 μg/kg/min), (e) LY344864 (5-HT_1F_; 20 μg/kg/min), or (f) AS-19 (5-HT_7_; 20 μg/kg/min). After 15 min of starting the corresponding infusion, MBP and HR were determined again, and the sensory CGRPergic outflow was electrically stimulated to elicit vasodepressor responses by applying 25-s trains of monophasic pulses (1 ms, 50 V) at increasing stimulation frequencies (0.1, 0.5, 1, and 5 Hz), as previously described (Cuesta et al., 2014). Once MBP returned to baseline, the next frequency was applied to obtain the S-R curve.

The second group (Group 2; male rats; n = 10) was pretreated with an i.v. bolus injection of SB699551 (5-HT_5A_ antagonist; 1 mg/kg). Five minutes later, an infusion of saline (1 mL/h; n = 5) or 5−CT (0.001 μg/kg/min; n = 5) was given; after 15 min, a S-R curve was constructed as described above.

The third group (Group 3; female rats; n = 35) was subdivided into seven subgroups (n = 5 each) comprising i.v. perfusions of: (a) 1 mL/h saline; (b) 1 mL/h EtOH 5%; (c) 0.001 μg/kg/min 5−CT; (d) 20 μg/kg/min 8-OH-DPAT (5-HT_1A_ agonist); (e) 20 μg/kg/min sumatriptan (5-HT_1B/1D_ agonist); (f) 20 μg/kg/min LY344864 and (g) 20 μg/kg/min AS-19. After 15 min, a S-R curve was obtained as described above.

The fourth group (Group 4; female rats; n = 20) received an i.v. bolus injection of SB699551 (5-HT_5A_ antagonist; 1 mg/kg) or a combination of GR127935 plus SB258719 (5-HT_1B/1D/1F_ antagonist; 310 μg/kg; and 5-HT_7_ antagonist; 1 mg/kg, respectively). Five minutes later, an infusion of saline (1 mL/h; n = 5 each) or 5−CT (0.001 μg/kg/min; n = 5 each) was given; after 15 min, a S-R curve was obtained as described above.

#### 2.3.2 Protocol II. Administration of exogenous α-CGRP

In the second set of rats (Figure 1; female group; n = 20) the pithing rod was left in place throughout the experiment, and neither d-tubocurarine nor hexamethonium was administered since no electrical stimulation was performed. All animals on this set received a continuous i.v. perfusion of methoxamine (20 μg/kg/min). After 25 min, when a stable haemodynamic condition was reached, this set was divided into four subgroups (n = 5 each) that received (i.v.): (i) saline (1 mL/h); (ii) EtOH 5% (1 mL/h); (iii) LY344864 (20 μg/kg/min); or (iv) AS-19 (20 μg/kg/min). Fifteen minutes later, MBP and HR were reassessed, and the vasodepressor responses induced by i.v. bolus administration of exogenous α-CGRP (0.1, 0.3 and 1 μg/kg) were examined during the infusion of vehicles or 5-HT agonists.

### 2.4 Statistical analysis and data presentation

All experimental protocols and data analysis were randomised and blinded. Results are presented as mean ± SEM of at least five experiments (n = 5). The changes in MBP (ΔMBP) by electrical stimulation or exogenous α-CGRP are represented as decreases in MBP from the baseline value. Statistical analyses were performed using GraphPad Prism 9.3.0 (GraphPad, USA). Normal distribution was determined using the Shapiro-Wilk test and homogeneity of variances was assessed by Brown-Forsythe test. Variations in basal MBP and HR before and after the corresponding treatment were evaluated by t-test with Welch correction. Moreover, vasodepressor responses obtained by electrical stimulation or exogenous α-CGRP in the different subgroups of animals were compared with a two-way ANOVA, followed by Dunnett’s (compared to control group) *post hoc* test. *Post hoc* tests were conducted only if F in ANOVA achieved P < 0.05. Statistical significance was accepted at P < 0.05. Since the decreases in MBP induced by electrical stimulation or i.v. α-CGRP in the presence of i.v. EtOH did not differ from those obtained with saline, statistical comparisons were performed only versus the saline group.

## 3 Results

### 3.1 Systemic haemodynamic variables

**FIGURE 2:**
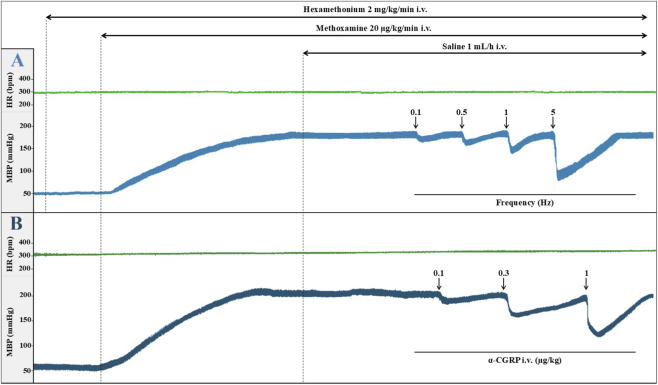
Original experimental tracings showing mean blood pressure (MBP; mmHg), heart rate (HR; beats/min, bpm) and vasodepressor responses induced by **(A)** electrical stimulation of the perivascular CGRPergic outflow or **(B)** i.v. bolus administration of α-CGRP, during continuous i.v. infusion of saline (1 mL/h) in female pithed rats.

After anaesthesia, basal MBP and HR in male pithed rats (n = 40) were 64 ± 2 mmHg and 319 ± 5 bpm, respectively, whereas in females (n = 75) these values were significantly lower compared with males: 53 ± 1 mmHg and 302 ± 5 bpm, respectively (*P < 0.05 vs. males). These variables were not modified after the i.v. bolus administration of atropine or d-tubocurarine (not shown), or during the continuous infusion of hexamethonium (69 ± 2 mmHg and 327 ± 6 bpm in males; 54 ± 1 mmHg and 306 ± 4 bpm in females). By contrast, methoxamine infusion (20 μg/kg/min, after 25 min) substantially increased both parameters, reaching 184 ± 4 mmHg and 362 ± 9 bpm in males, and 183 ± 2 mmHg and 335 ± 5 bpm in females (*P < 0.05 vs. the corresponding basal value) (Figure 2). Interestingly, the methoxamine-induced increase in MBP was significantly greater in females (127.9 ± 2.2 mmHg) compared with males (118.7 ± 3.5 mmHg) (*P < 0.05 vs. males).

During methoxamine infusion, MBP and HR were not altered by i.v. perfusion/bolus of 5-HT agonists/antagonists (or their vehicles) in either male or female pithed rats (Figure 2; Table 1). The only exception, observed in both sexes, was a sustained decrease in MBP induced by the perfusion of 5−CT (0.001 μg/kg/min) and AS-19 (20 μg/kg/min) (Table 1). Moreover, in female pithed rats, the vasodilator effect of 5−CT was reversed by pretreatment with the mixture of GR127935 plus SB258719 (ΔMBP: −13.2 ± 1.8 mmHg; *P < 0.05 vs. 5−CT, P > 0.05 vs. saline).

**TABLE 1:** Changes in mean blood pressure (ΔMBP; mmHg) and heart rate (ΔHR; bpm) 15 min after starting saline or 5-HT agonist perfusion in pithed rats under methoxamine infusion (20 μg/kg/min).

Wistar Rat	Treatment	Dose (µg/kg/min)	ΔMBP (mmHg)	ΔHR (bpm)
Males	Saline	1 mL/h	2.3 ± 5.7	13.0 ± 1.5
	EtOH 5%	1 mL/h	−3.8 ± 0.6	6.8 ± 2.5
	5-CT	0.001	−38.2 ± 2.9*	5.6 ± 4.6
	AS-19	20	−47.8 ± 14.1*	11.3 ± 13.5
	CP-93,129	20	−14.1 ± 5.4	18.0 ± 3.1
	LY344864	20	−1.2 ± 2.8	7.7 ± 4.3
Females	Saline	1 mL/h	−0.4 ± 0.3	5.6 ± 3.2
	EtOH 5%	1 mL/h	−5.6 ± 3.9	7.7 ± 7.9
	5-CT	0.001	−36.7 ± 6.8*	7.0 ± 3.5
	AS-19	20	−44.0 ± 5.6*	15.3 ± 1.8
	8-OH-DPAT	20	−2.3 ± 3.9	7.4 ± 4.5
	Sumatriptan	20	−0.3 ± 2.0	3.6 ± 6.0
	LY344864	20	−7.2 ± 4.1	6.7 ± 7.5

All values are expressed as mean ± SEM (n = 5). Data were analysed by t-test with Welch correction. *P < 0.05 vs. baseline. bpm: beats/min.

### 3.2 Vascular responses by sensory nerves stimulation in male and female rats

During the continuous infusion of methoxamine, which produced a sustained increase in vascular tone throughout the experiment, electrical stimulation of the perivascular sensory outflow (0.1–5 Hz) resulted in frequency-dependent decreases in MBP in male and female pithed rats (Figure 2A) (Cuesta et al., 2014). In all cases, these vasodepressor responses were due to selective systemic vasodilation, since (i) only minor variations were observed in HR and (ii) appeared about 10 s after the stimulus and reached a maximum 1 min after the stimulus has ended (Cuesta et al., 2014; González-Hernández et al., 2010; 2011; Figure 2).

**FIGURE 3:**
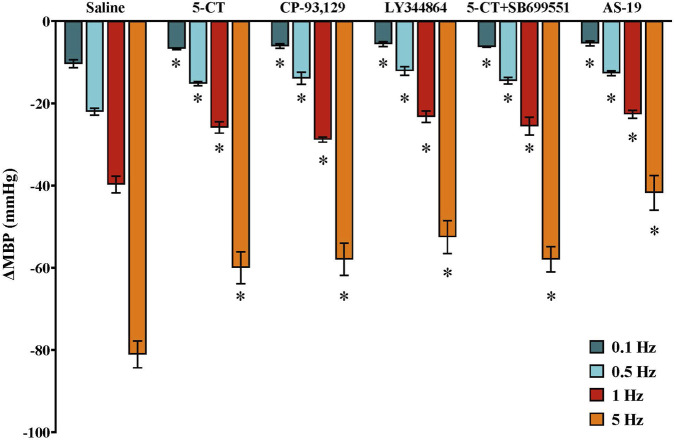
Effect of continuous i.v. perfusion of saline (1 mL/h; control), 5−CT (0.001 μg/kg/min), CP-93,129 (20 μg/kg/min), LY344864 (20 μg/kg/min), 5−CT (0.001 μg/kg/min) in the presence of SB699551 (1 mg/kg i.v.) or AS-19 (20 μg/kg/min) (n = 5 each) on the vasodepressor responses induced by electrical stimulation of the CGRPergic outflow in male rats. Data were analysed by two-way ANOVA followed by Dunnett’s *post hoc* test. All values are expressed as mean ± SEM. *P < 0.05 vs. saline. ΔMBP: changes in mean blood pressure.

**FIGURE 4:**
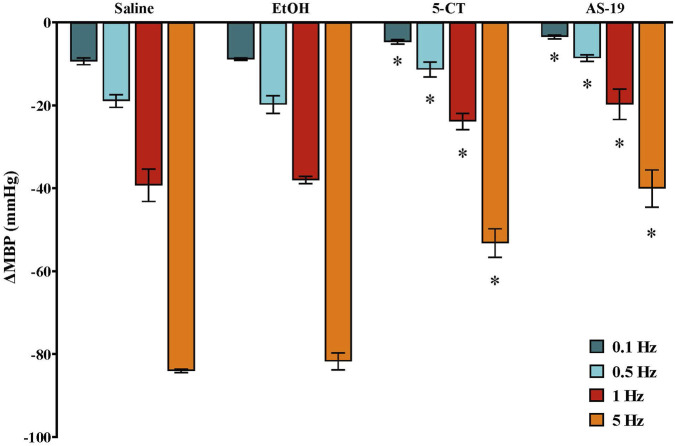
Effect of continuous i.v. perfusion of saline (1 mL/h; control), ethanol 5% (EtOH, 1 mL/h), 5−CT (0.001 μg/kg/min) or AS-19 (20 μg/kg/min) (n = 5 each) on the vasodepressor responses induced by electrical stimulation of the CGRPergic outflow in female rats. Data were analysed by two-way ANOVA followed by Dunnett’s *post hoc* test. All values are expressed as mean ± SEM. *P < 0.05 vs. saline. ΔMBP: changes in mean blood pressure.

**FIGURE 5:**
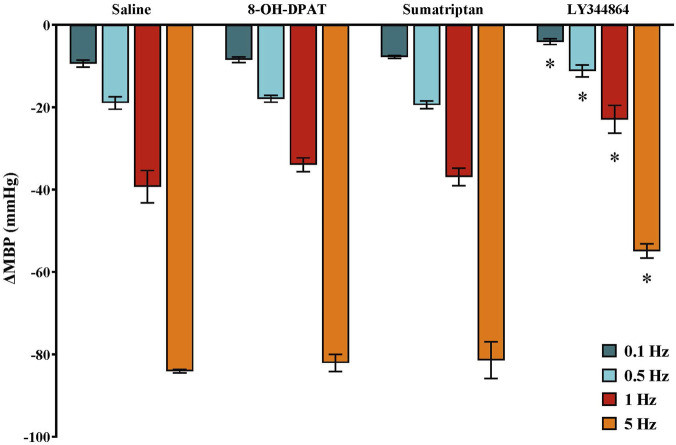
Effect of continuous i.v. perfusion of saline (1 mL/h; control), 8-OH-DPAT, sumatriptan or LY344864 (20 μg/kg/min and n = 5 each) on the vasodepressor responses induced by electrical stimulation of the CGRPergic outflow in female rats. Data were analysed by two-way ANOVA followed by Dunnett’s *post hoc* test. All values are expressed as mean ± SEM. *P < 0.05 vs. saline. ΔMBP: changes in mean blood pressure.

**FIGURE 6:**
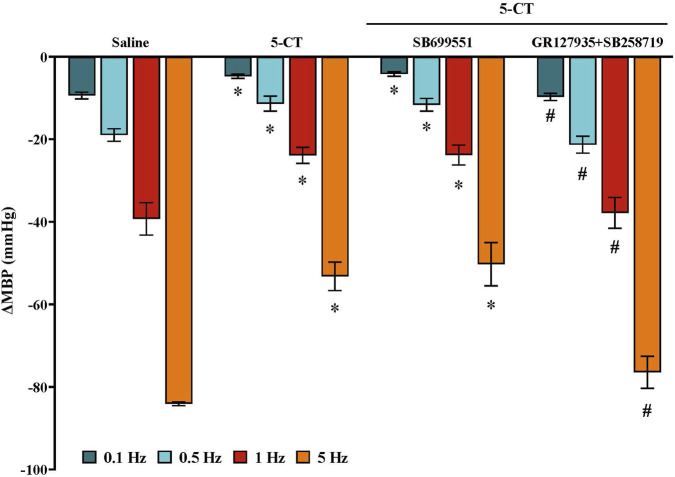
Effect of continuous i.v. perfusion of 5−CT (0.001 μg/kg/min) alone or in the presence of SB699551 (1 mg/kg i.v.) or a cocktail of GR127935 (310 μg/kg i.v.) + SB258719 (1 mg/kg i.v.) on the vasodepressor responses induced by electrical stimulation of the CGRPergic outflow in female rats. Data were analysed by two-way ANOVA followed by Dunnett’s *post hoc* test. All values are expressed as mean ± SEM. *P < 0.05 vs. saline; #P < 0.05 vs. 5−CT alone. ΔMBP: changes in mean blood pressure.

In male pithed rats, vasodepressor responses induced by electrical stimulation (S-R curve) during continuous i.v. saline perfusion (control; 1 mL/h) were −10.4 ± 1.0, −22.0 ± 0.9, −39.7 ± 2.0 and −81.1 ± 3.3 mmHg (Figure 3). In females, the corresponding values were −9.4 ± 0.8, −19.0 ± 1.5, −39.3 ± 3.9 and −84.1 ± 0.5 mmHg (Figures 4–6), with no significant differences between sexes.

### 3.3 Effect of ethanol and 5-HT receptor ligands on the vasodepressor responses induced by electrical stimulation in male rats

In male pithed rats, the decreases in MBP due to electrical stimulation were not modified by i.v. infusion of EtOH 5% (1 mL/h), as previously reported (Cuesta et al., 2014). In contrast, continuous infusion of 5−CT (0.001 μg/kg/min), a selective 5-HT_1/5/7_ receptor agonist, reduced the electrically induced vasodepressor responses (Figure 3). This inhibitory effect was mimicked by i.v. infusion of CP-93,129 (5-HT_1B_ agonist), LY344864 (5-HT_1F_ agonist) and AS-19 (5-HT_7_ agonist), each at dose of 20 μg/kg/min (Figure 3).

The i.v. pretreatment with the selective 5-HT_5A_ receptor subtype antagonist, SB699551 (1 mg/kg), did not affect the vasodepressor responses induced by electrical stimulation in male rats (data not shown). Furthermore, the inhibitory effect of 5−CT (0.001 μg/kg/min) on these responses was not altered by pretreatment with SB699551 (Figure 3).

### 3.4 Effect of ethanol and 5-HT receptor type/subtype agonists on the electrically induced vasodepressor responses in female rats

Continuous infusion of EtOH 5% (1 mL/h) did not affect the vasodepressor responses induced by electrical stimulation in female pithed rats (Figure 4). In contrast, i.v. perfusion of 5−CT (5-HT_1/5/7_ agonist; 0.001 μg/kg/min) and AS-19 (5-HT_7_ agonist; 20 μg/kg/min) inhibited the vascular CGRPergic outflow (Figure 4).

To investigate the involvement of 5-HT_1_ receptor subtypes in this inhibitory effect, the agonists tested (all at 20 μg/kg/min) were: 8-OH-DPAT (5-HT_1A_ agonist), sumatriptan (5-HT_1B/1D_ agonist) and LY344864 (5-HT_1F_ agonist). Only LY344864 significantly reduced the electrically induced vasodepressor responses, whereas 8-OH-DPAT and sumatriptan had no effect (Figure 5).

### 3.5 Influence of 5-HT receptor antagonists on 5-CT-induced vascular sensory-inhibition in female rats

During an i.v. infusion of the vehicle of 5−CT (saline; 1 mL/h), the electrically induced vasodepressor responses in females receiving i.v. pretreatment with (i) SB699551 (5-HT_5A_ antagonist; 1 mg/kg) or (ii) a combination of GR127935 plus SB258719 (5-HT_1B/1D/1F_ antagonist; 310 μg/kg; and 5-HT_7_ antagonist; 1 mg/kg, respectively) remained unaltered (data not shown). The 5-CT-induced sensory inhibition was (i) unaffected by SB699551 (1 mg/kg) and (ii) abolished by the mixture of GR127935 plus SB258719 (310 μg/kg and 1 mg/kg, respectively) (Figure 6).

### 3.6 Effect of ethanol and selective 5-HT agonists on the vasodepressor responses induced by i.v. administration of α-CGRP in female rats

The i.v. administration of exogenous α-CGRP (0.1–1 μg/kg) caused dose-dependent decreases in MBP with minor changes in HR (Figure 2B; Table 2). Neither EtOH 5% (1 mL/h) nor LY344864 (5-HT_1F_ agonist; 20 μg/kg/min) modified these vasodepressor responses (Table 2). In contrast, AS-19 (5-HT_7_ agonist; 20 μg/kg/min) significantly reduced the i.v. CGRP-induced vasodepressor responses (Table 2).

**TABLE 2:** Effect of continuous i.v. infusion of ethanol 5% (EtOH, 1 mL/h), LY344864 and AS-19 (20 μg/kg/min each 5-HT agonist) on the vasodepressor responses induced by i.v. administration of α-CGRP (0.1, 0.3 and 1 μg/kg) in female pithed rats.

Treatment	α-CGRP 0.1 μg/kg	α-CGRP 0.3 μg/kg	α-CGRP 1.0 μg/kg
Control (saline)	−17.4 ± 0.7	−37.9 ± 0.8	−71.7 ± 3.8
EtOH 5%	−17.1 ± 0.3	−36.8 ± 0.8	−72.7 ± 0.5
LY344864	−19.9 ± 1.6	−41.2 ± 1.0	−78.7 ± 3.7
AS-19	−10.2 ± 1.2*	−19.1 ± 2.3*	−38.1 ± 5.4*
Measurement	ΔMBP (mmHg)		

All values are expressed as mean ± SEM (n = 5). Data were analysed by two-way ANOVA followed by Dunnett’s *post hoc* test. *P < 0.05 vs. control. ΔMBP: changes in mean blood pressure.

## 4 Discussion

The maintenance of BP depends on the dynamic balance between peripheral vascular tone and cardiac output, which are regulated by neuronal, humoral and local mechanisms. In this sense, the serotonergic system modulates vascular sympathetic and sensory neurotransmission, and both are influenced by sex steroid hormones (Cuesta et al., 2014; Allais et al., 2020; García-Pedraza et al., 2021; 2022; Fernández-González et al., 2022; 2023; 2024; González-Hernández et al., 2023; Terol-Úbeda et al., 2025a; 2025b). In the present study, we show for the first time that peripheral 5-HT receptors modulate perivascular sensory CGRPergic outflow in female rats. Prejunctional 5-HT_1F_ receptors and pre and/or postjunctional 5-HT_7_ receptors inhibited the perivascular CGRPergic neurotransmission. These findings extend previous evidence on serotonergic modulation of vascular function and support the existence of sex-dependent mechanisms in cardiovascular regulation.

There is strong evidence that BP regulation is influenced by biological effects of sex chromosomes, sex hormones and reproductive events (Gerdts et al., 2022). During the fertile period, females have lower BP than age-matched males, mainly due to the reproductive hormone oestradiol, which downregulates vascular sympathetic nerve activity, attenuates calcium signaling in vascular, renal and cardiac cells, and controls the synthesis of potent vasoconstrictors, such as angiotensin II (Gerdts et al., 2022). In accordance with this, and as previously demonstrated (Terol-Úbeda et al., 2025b), in our experimental model of 14–16-week-old female (cycling period) anaesthetised pithed rats (with no central influence on BP), MBP and HR were significantly lower (around 10 mmHg and 10 bpm, respectively) compared with male rats.

As previously established in pithed rats to induce vasodepressor responses, MBP was sustainedly elevated by a methoxamine infusion. Methoxamine enhances peripheral vascular resistance via α_1_-adrenoreceptor activation, while the increase in HR is mediated through cardiac α_1_-adrenoreceptor activation (González-Hernández et al., 2011; Cuesta et al., 2014). In this context, the methoxamine-induced increase in MBP was higher in female than in male rats. In line with these findings, previous evidence indicates sex-dependent differences in α- and β-adrenergic responsiveness across several vascular territories (Passmore et al., 2005; Alexandre et al., 2017; Sherwood et al., 2017), which may contribute to the enhanced pressor effect observed in females.

It is noteworthy that, in both male and female rats, the attenuation of the methoxamine-induced increase in MBP produced by the infusion of 5−CT (0.001 μg/kg/min) or AS-19 (20 μg/kg/min) likely reflects the activation of vasorelaxant 5-HT_1/5/7_ or 5-HT_7_ receptors, respectively, leading to a long-lasting vasodilation. Previous studies from our group demonstrated 5-HT_7_ receptor-mediated vasodilation in male pithed rats under comparable experimental conditions (Cuesta et al., 2014). Similarly, intravenous infusion of 5−CT decreased MBP in both male (García-Pedraza et al., 2021) and female (Terol-Úbeda et al., 2025b) pithed rats without methoxamine. In addition, several pharmacological observations support the involvement of 5-HT_7_ receptors in the vasodilator effect of 5−CT in female rats: (i) 5-CT-induced vasodilation was abolished by pretreatment with GR127935 plus SB258719 (5-HT_1B/1D/1F_ and 5-HT_7_ receptor antagonist, respectively); (ii) selective activation of 5-HT_1B/1D_ and 5-HT_1F_ receptors did not modify the MBP values during methoxamine infusion; and (iii) blockade of 5-HT_5A_ receptors failed to affect 5-CT-induced vasodilation. Together, these findings support that the vasodilator effect of 5−CT in female rats is mainly mediated by 5-HT_7_ receptors, as previously shown in males (Cuesta et al., 2014).

Under the presence of hexamethonium (to block autonomic outflow) and methoxamine, electrical stimulation of perivascular sensory CGRPergic nerves elicited frequency-dependent vasodepressor responses in both male and female rats, without significant variations in HR. Similarly, decreases in MBP induced by intravenous administration of exogenous α-CGRP in female rats were dose-dependent, as previously described in males (Cuesta et al., 2014). The inhibition of the electrically induced vasodepressor responses by the 5-HT_1/5/7_ receptor agonist 5−CT, observed in both male and female rats, demonstrates that the serotonergic system modulates perivascular sensory CGRPergic neurotransmission. In male rats, this 5-CT-mediated inhibition is consistent with previous findings showing that activation of prejunctional 5-HT_1B/1F_ and 5-HT_7_ receptors reduces CGRP release from perivascular sensory nerves (González-Hernández et al., 2010; 2011; Cuesta et al., 2014). At the prejunctional level, 5-HT can modulate the release of autonomic and sensory neurotransmitters, including noradrenaline, acetylcholine and CGRP, facilitating hypertensive or hypotensive effects (Marichal-Cancino et al., 2020; Fernández-González et al., 2022; 2023; 2024; González-Hernández et al., 2023; Terol-Ubeda et al., 2025a; Terol-Ubeda et al., 2025b). In both male and female rats, 5−CT inhibits the vasoconstriction induced by sympathetic stimulation, involving 5-HT_1D/1A_ receptor subtypes in males whereas in females only 5-HT_1D_ receptors are implicated (Morán et al., 1994; García et al., 2005; García-Pedraza et al., 2013; García-Pedraza et al., 2021; Terol-Úbeda et al., 2025a; Terol-Úbeda et al., 2025b).

Given this sex-dependent serotonergic regulation of vascular function, it is plausible that hormonal influences contribute to differences in 5-HT-mediated modulation of perivascular sensory neurotransmission. Oestrogens regulate the gene expression of 5-HT receptors, promote 5-HT synthesis by increasing tryptophan hydroxylase activity, and reduce 5-HT degradation and reuptake through modulation of monoamine oxidases and the 5-HT reuptake transporter, respectively (Nappi et al., 2022). Moreover, oestrogen, 5-HT and CGRP receptors coexist within the trigeminovascular system (Aggarwal et al., 2012), supporting a functional link between serotonergic and CGRPergic pathways in migraine pathophysiology, a female-predominant disorder. Women experience more frequent, longer, and severe migraine attacks than men, and although serotonergic antimigraine drugs have demonstrated clinical efficacy in both sexes, most trials lack sufficient statistical power to assess potential sex-related differences in therapeutic response (Elgendy et al., 2019; de Vries et al., 2020). These considerations guided the present investigation to identify the 5-HT receptor (sub)types involved in the 5-CT-induced inhibition of perivascular sensory CGRPergic outflow in female rats.

The receptor (sub)types involved in the 5-CT-induced inhibition of perivascular CGRPergic outflow were first confirmed in male rats, and the role of 5-HT_5A_ receptors was explored, as their contribution had not been previously investigated. Activation of 5-HT_1B_, 5-HT_1F_, and 5-HT_7_ receptors, using the selective agonists CP-93,129, LY344864, and AS-19, respectively, inhibited the vasodepressor responses induced by sensory CGRPergic stimulation, as previously described (González-Hernandez et al., 2010; 2011; Cuesta et al., 2014). Although 5-HT_5A_ receptors were excluded in the present study, given that 5−CT maintained its inhibitory action in the presence of the 5-HT_5A_ receptor antagonist SB699551, these receptors have been implicated in autonomic cardiovascular regulation, particularly in the inhibition of cardioaccelerator sympathetic outflow under specific experimental conditions in male rats (García-Pedraza et al., 2018; García-Pedraza et al., 2020). In female rats, the inhibitory effect of 5−CT on the vasodepressor responses induced by CGRPergic stimulation was reproduced by 5-HT_1F_ and 5-HT_7_ receptor agonists (LY344864 and AS-19, respectively), whereas neither 5-HT_1A_ nor 5-HT_1B/1D_ receptor agonists (8-OH-DPAT and sumatriptan, respectively) modified the electrically induced vasodilations. As in males, 5-HT_5A_ receptors were unlikely to contribute to the 5-CT-mediated inhibition of the vasodepressor sensory CGRPergic drive, as this effect persisted after SB699551 administration. Consistent with this, previous studies reported no role for 5-HT_5A_ receptors in the serotonergic modulation of vascular sympathetic outflow in female rats (Terol-Úbeda et al., 2025b). The participation of 5-HT_1F_ and 5-HT_7_ receptors in females was further confirmed using a mixture of GR127935 plus SB258719, which completely reversed the 5-CT-induced inhibition of perivascular sensory CGRPergic outflow. Altogether, these results highlight the sex-dependent serotonergic inhibition of perivascular sensory CGRPergic innervation, as in male rats 5-HT_1F_ and 5-HT_7_, but also 5-HT_1B_, receptors were involved as inhibitors of neurovascular CGRPergic drive (González-Hernández et al., 2010; González-Hernández et al., 2011; Cuesta et al., 2014).

To clarify the nature of the 5-HT receptors involved in the inhibition of CGRPergic vascular responses in female rats, we determined whether LY344864 or AS-19 modified the vasodepressor responses to exogenous α-CGRP. Although other effects (as altered vascular responsiveness to CGRP) cannot be fully excluded, we conclude that 5-HT_1F_ receptor-mediated inhibition was prejunctional in nature, whereas 5-HT_7_ receptors were pre and/or postjunctional in nature, since AS-19 also decreased the vasodepressor responses induced by exogenous α-CGRP. The latter differs from results in male rats, where 5-HT_7_ receptors implicated in serotonergic inhibition of perivascular sensory CGRPergic outflow were purely prejunctional (Cuesta et al., 2014).

Although previous studies have reported sex-related differences in the serotonergic modulation of vascular sympathetic neurotransmission, affecting both the receptor (sub)types involved and their pre and/or postjunctional localization (Terol-Úbeda et al., 2025b), evidence of sexual dimorphism in peripheral 5-HT_1_ and 5-HT_7_ receptor function has remained limited (Spies et al., 2020). The present findings in female rats further illustrate the complexity of the serotonergic system, revealing a novel peripheral, sex-dependent mechanism in the modulation of perivascular CGRPergic responses. Sexual dimorphism influences not only the 5-HT receptors involved in the inhibition of the vasodepressor responses induced by sensory CGRPergic stimulation, but also the nature of these receptors.

Some limitations of the present study should be considered. Experiments were performed under pithed condition, thus excluding any influence of the CNS on cardiovascular regulation, and therefore the central component of migraine is not studied. The experimental design did not allow determination of the mechanisms underlying tachyphylaxis of the CGRPergic vasodepressor responses. Moreover, sensory nerve activity was not recorded directly; instead, electrically evoked CGRP release in the systemic vasculature was inferred from the magnitude of the vasodepressor responses (Cuesta et al., 2014). Additionally, this study did not assess ovarian hormones or the estrous cycle stage of female rats, since we only considered animals of reproductive age. Anyhow, our present results contribute to a better understanding of sex-related differences in basic cardiovascular pharmacology, a fundamental aspect for developing effective therapeutic strategies for both women and men with cardiovascular diseases (CVD). As several antimigraine therapies, including triptans and ditans, target serotonergic and CGRP-related pathways (Viticchi et al., 2022; Kalkman et al., 2023), and patients with migraine exhibit a higher incidence of CVD (Linstra et al., 2017; Rajendran et al., 2023), clarifying sex-specific serotonergic-CGRPergic interactions is essential to improve therapeutic efficacy and minimize cardiovascular adverse effects. Given that CVD remain the leading cause of death among women worldwide (Vervoort et al., 2024), and migraine represents a major vascular risk factor in females, our findings reinforce the importance of conducting preclinical pharmacological studies in both male and female animal models to better predict sex-specific cardiovascular responses.

In conclusion, the present study demonstrates that 5−CT inhibits perivascular sensory CGRPergic neurotransmission in both male and female rats. Unlike males, where the 5−CT effect is mediated by prejunctional 5-HT_1B/1F/7_ receptors, in cycling-age female rats this inhibitory effect involves prejunctional 5-HT_1F_ and pre and/or postjunctional 5-HT_7_ receptors. These findings reveal a sex-dependent serotonergic modulation of vascular CGRPergic function, highlighting its potential relevance in neurovascular pharmacology.

## Data Availability

The raw data supporting the conclusions of this article will be made available by the authors, without undue reservation.

## References

[B1] Aggarwal M. Puri V. Puri S. (2012). Serotonin and CGRP in migraine. Ann. Neurosci. 19 (2), 88–94. 10.5214/ans.0972.7531.12190210 25205974 PMC4117050

[B2] Alexandre E. C. de Oliveira M. G. Campos R. Kiguti L. R. Calmasini F. B. Silva F. H. (2017). How important is the α_1_-adrenoceptor in primate and rodent proximal urethra? Sex differences in the contribution of α_1_-adrenoceptor to urethral contractility. Am. J. Physiol. Ren. Physiol. 312 (6), F1026–F1034. 10.1152/ajprenal.00013.2017 28298357

[B3] Allais G. Chiarle G. Sinigaglia S. Airola G. Schiapparelli P. Benedetto C. (2020). Gender-related differences in migraine. Neurol. Sci. 41 (Suppl. 2), 429–436. 10.1007/s10072-020-04643-8 32845494 PMC7704513

[B4] Barnes N. M. Ahern G. P. Becamel C. Bockaert J. Camilleri M. Chaumont-Dubel S. (2021). International union of basic and clinical pharmacology. CX. Classification of receptors for 5-hydroxytryptamine; pharmacology and function. Pharmacol. Rev. 73 (1), 310–520. 10.1124/pr.118.015552 33370241 PMC7770494

[B5] Bonura A. Brunelli N. Marcosano M. Iaccarino G. Fofi L. Vernieri F. (2023). Calcitonin gene-related peptide systemic effects: embracing the complexity of its biological Roles-A narrative review. Int. J. Mol. Sci. 24 (18), 13979. 10.3390/ijms241813979 37762283 PMC10530509

[B6] Cuesta C. García-Pedraza J. Á. García M. Villalón C. M. Morán A. (2014). Role of 5-HT_7_ receptors in the inhibition of the vasodepressor sensory CGRPergic outflow in pithed rats. Vasc. Pharmacol. 63 (1), 4–12. 10.1016/j.vph.2014.06.009 25179247

[B7] de Vries T. Villalón C. M. MaassenVanDenBrink A. (2020). Pharmacological treatment of migraine: CGRP and 5-HT beyond the triptans. Pharmacol. Ther. 211, 107528. 10.1016/j.pharmthera.2020.107528 32173558

[B8] de Vries Lentsch S. Rubio-Beltrán E. MaassenVanDenBrink A. (2021). Changing levels of sex hormones and calcitonin gene-related peptide (CGRP) during a woman's life: implications for the efficacy and safety of novel antimigraine medications. Maturitas 145, 73–77. 10.1016/j.maturitas.2020.12.012 33541566

[B9] Dearing C. Handa R. J. Myers B. (2022). Sex differences in autonomic responses to stress: implications for cardiometabolic physiology. Am. J. Physiol. Endocrinol. Metab. 323 (3), E281–E289. 10.1152/ajpendo.00058.2022 35793480 PMC9448273

[B10] Elgendy I. Y. Nadeau S. E. Bairey Merz C. N. Pepine C. J. (2019). Migraine headache: an under-appreciated risk factor for cardiovascular disease in women. J. Am. Heart Assoc. 8, e014546. 10.1161/JAHA.119.014546 31707945 PMC6915301

[B11] Favoni V. Giani L. Al-Hassany L. Asioli G. M. Butera C. de Boer I. (2019). CGRP and migraine from a cardiovascular point of view: what do we expect from blocking CGRP? J. Headache Pain. 20 (1), 27. 10.1186/s10194-019-0979-y 30866804 PMC6734543

[B12] Feng Y. Li X. Li Z. He X. Tang Y. Tian W. (2025). The effects of 5-HT on vascular endothelial dysfunction in patients with panic disorder. Front. Cardiovasc. Med. 12, 1632070. 10.3389/fcvm.2025.1632070 40910147 PMC12405210

[B13] Fernández-González J. F. García-Pedraza J. Á. Marín-Quílez A. Bastida J. M. Martín M. L. Morán A. (2022). Effect of sarpogrelate treatment on 5-HT modulation of vascular sympathetic innervation and platelet activity in diabetic rats. Biomed. Pharmacother. 153, 113276. 10.1016/j.biopha.2022.113276 35717784

[B14] Fernández-González J. F. García-Pedraza J. Á. Ordóñez J. L. Terol-Úbeda A. C. Martín M. L. Morán A. (2023). Renal sympathetic hyperactivity in diabetes is modulated by 5-HT_1D_ receptor activation *via* NO pathway. Int. J. Mol. Sci. 24 (2), 1378. 10.3390/ijms24021378 36674892 PMC9865738

[B15] Fernández-González J. F. García-Pedraza J. Á. Terol-Úbeda A. C. Martín M. L. Morán A. García-Domingo M. (2024). Chronic sarpogrelate treatment improves renal sympathetic hyperactivity in experimental diabetes. Biomed. Pharmacother. 176, 116814. 10.1016/j.biopha.2024.116814 38820974

[B16] Ferrari M. D. Goadsby P. J. Burstein R. Kurth T. Ayata C. Charles A. (2022). Migraine. Nat. Rev. Dis. Prim. 8 (1), 2. 10.1038/s41572-021-00328-4 35027572

[B17] García M. Morán A. Calama E. Martín M. L. Barthelmebs M. Román L. S. (2005). Diabetes-induced changes in the 5-hydroxytryptamine inhibitory receptors involved in the pressor effect elicited by sympathetic stimulation in the pithed rat. Br. J. Pharmacol. 145 (5), 593–601. 10.1038/sj.bjp.0706216 15852039 PMC1576173

[B18] García-Pedraza J. Á. García M. Martín M. L. Gómez-Escudero J. Rodríguez-Barbero A. Román L. S. (2013). Peripheral 5-HT_1D_ and 5-HT_7_ serotonergic receptors modulate sympathetic neurotransmission in chronic sarpogrelate treated rats. Eur. J. Pharmacol. 714 (1-3), 65–73. 10.1016/j.ejphar.2013.05.045 23769743

[B19] García-Pedraza J. Á. Hernández-Abreu O. García M. Morán A. Villalón C. M. (2018). Chronic 5-HT_2_ receptor blockade unmasks the role of 5-HT_1F_ receptors in the inhibition of rat cardioaccelerator sympathetic outflow. Can. J. Physiol. Pharmacol. 96 (4), 328–336. 10.1139/cjpp-2017-0191 28886249

[B20] García-Pedraza J. Á. Hernández-Abreu O. Morán A. Carretero J. García-Domingo M. Villalón C. M. (2020). Role of peripheral 5-HT_5A_ receptors in 5-HT-induced cardiac sympatho-inhibition in type 1 diabetic rats. Sci. Rep. 10 (1), 19358. 10.1038/s41598-020-76298-6 33168874 PMC7652863

[B21] García-Pedraza J. Á. López C. Fernández-González J. F. Martín M. L. Morán A. García-Domingo M. (2021). Vascular sympathetic neurotransmission and its serotonergic regulation are modified by chronic fluoxetine treatment. J. Pharmacol. Sci. 147 (1), 48–57. 10.1016/j.jphs.2021.05.008 34294372

[B22] García-Pedraza J. Á. Fernández-González J. F. López C. Martín M. L. Alarcón-Torrecillas C. Rodríguez-Barbero A. (2022). Oral fluoxetine treatment changes serotonergic sympatho-regulation in experimental type 1 diabetes. Life Sci. 293, 120335. 10.1016/j.lfs.2022.120335 35051421

[B23] GBD 2021 Headache Collaborators Wijeratne T. Oh J. Kim S. Yim Y. Kim M. S. (2025). Global, regional, and national burden of headache disorders, 1990-2021, with forecasts to 2050: a global Burden of disease study 2021. Cell. Rep. Med. 6 (10), 102348. 10.1016/j.xcrm.2025.102348 40972580 PMC12629824

[B24] Gerdts E. Sudano I. Brouwers S. Borghi C. Bruno R. M. Ceconi C. (2022). Sex differences in arterial hypertension. Eur. Heart J. 43 (46), 4777–4788. 10.1093/eurheartj/ehac470 36136303 PMC9726450

[B25] González-Hernández A. Muñoz-Islas E. Lozano-Cuenca J. Ramírez-Rosas M. B. Sánchez-López A. Centurión D. (2010). Activation of 5-HT_1B_ receptors inhibits the vasodepressor sensory CGRPergic outflow in pithed rats. Eur. J. Pharmacol. 637 (1-3), 131–137. 10.1016/j.ejphar.2010.03.053 20385119

[B26] González-Hernández A. Manrique-Maldonado G. Lozano-Cuenca J. Muñoz-Islas E. Centurión D. Maassen VanDenBrink A. (2011). The 5-HT_1_ receptors inhibiting the rat vasodepressor sensory CGRPergic outflow: further involvement of 5-HT_1F_, but not 5-HT_1A_ or 5-HT_1D_, subtypes. Eur. J. Pharmacol. 659 (2-3), 233–243. 10.1016/j.ejphar.2011.03.035 21473863

[B27] González-Hernández A. Marichal-Cancino B. A. Lozano-Cuenca J. López-Canales J. S. Muñoz-Islas E. Ramírez-Rosas M. B. (2016). Heteroreceptors modulating CGRP release at neurovascular junction: potential therapeutic implications on some vascular-related diseases. Biomed. Res. Int. 2016, 2056786. 10.1155/2016/2056786 28116293 PMC5223010

[B28] González-Hernández A. Marichal-Cancino B. A. MaassenVanDenBrink A. Villalón C. M. (2023). Serotonergic modulation of neurovascular transmission: a focus on prejunctional 5-HT receptors/mechanisms. Biomedicines 11 (7), 1864. 10.3390/biomedicines11071864 37509503 PMC10377335

[B29] Hay D. L. Walker C. S. (2017). CGRP and its receptors. Headache 57 (4), 625–636. 10.1111/head.13064 28233915

[B30] Kalkman D. N. Couturier E. G. M. El Bouziani A. Dahdal J. Neefs J. Woudstra J. (2023). Migraine and cardiovascular disease: what cardiologists should know. Eur. Heart. J. 44 (30), 2815–2828. 10.1093/eurheartj/ehad363 37345664

[B31] Krause D. N. Warfvinge K. Haanes K. A. Edvinsson L. (2021). Hormonal influences in migraine - interactions of oestrogen, oxytocin and CGRP. Nat. Rev. Neurol. 17 (10), 621–633. 10.1038/s41582-021-00544-2 34545218

[B32] Lewter L. A. Arnold R. L. Narosov N. B. Dussor G. Kolber B. J. (2025). Sex differences in the effects of calcitonin gene-related peptide signaling on migraine-like behavior in animal models: a narrative review. Front. Neurol. 16, 1603758. 10.3389/fneur.2025.1603758 40708951 PMC12288688

[B33] Linstra K. M. Ibrahimi K. Terwindt G. M. Wermer M. J. MaassenVanDenBrink A. (2017). Migraine and cardiovascular disease in women. Maturitas 97, 28–31. 10.1016/j.maturitas.2016.12.008 28159058

[B34] Lozano-Cuenca J. González-Hernández A. Muñoz-Islas E. Sánchez-López A. Centurión D. Cobos-Puc L. E. (2009). Effect of some acute and prophylactic antimigraine drugs on the vasodepressor sensory CGRPergic outflow in pithed rats. Life Sci. 84 (5-6), 125–131. 10.1016/j.lfs.2008.11.008 19041880

[B35] Maddahi A. Warfvinge K. Holm A. Edvinsson J. C. A. Reducha P. V. Kazantzi S. (2023). Progesterone distribution in the trigeminal system and its role to modulate sensory neurotransmission: influence of sex. J. Headache Pain. 24 (1), 154. 10.1186/s10194-023-01687-x 37957603 PMC10644471

[B36] Marichal-Cancino B. A. González-Hernández A. Muñoz-Islas E. Villalón C. M. (2020). Monoaminergic receptors as modulators of the perivascular sympathetic and sensory CGRPergic outflows. Curr. Neuropharmacol. 18 (9), 790–808. 10.2174/1570159X18666200503223240 32364079 PMC7569320

[B37] Miguel-Martínez A. D. Linares-Bedolla J. Villanueva-Castillo B. Haanes K. A. MaassenVanDenBrink A. Villalón C. M. (2023). Pharmacological profile of the purinergic P2Y receptors that modulate, in response to ADPβS, the vasodepressor sensory CGRPergic outflow in pithed rats. Pharm. (Basel) 16 (3), 475. 10.3390/ph16030475 PMC10056196 36986572

[B38] Morán A. Velasco C. Salvador T. Martín M. L. San Román L. (1994). Inhibitory 5-hydroxytryptamine receptors involved in pressor effects obtained by stimulation of sympathetic outflow from spinal cord in pithed rats. Br. J. Pharmacol. 113 (4), 1358–1362. 10.1111/j.1476-5381.1994.tb17147.x 7889292 PMC1510480

[B39] Nappi R. E. Tiranini L. Sacco S. De Matteis E. De Icco R. Tassorelli C. (2022). Role of estrogens in menstrual migraine. Cells 11 (8), 1355. 10.3390/cells11081355 35456034 PMC9025552

[B40] Nichols D. E. Nichols C. D. (2008). Serotonin receptors. Chem. Rev. 108 (5), 1614–1641. 10.1021/cr078224o 18476671

[B41] Passmore J. C. Joshua I. G. Rowell P. P. Tyagi S. C. Falcone J. C. (2005). Reduced alpha adrenergic mediated contraction of renal preglomerular blood vessels as a function of gender and aging. J. Cell. Biochem. 96 (4), 672–681. 10.1002/jcb.20581 16149078

[B43] Pleş H. Florian I. A. Timis T. L. Covache-Busuioc R. A. Glavan L. A. Dumitrascu D. I. (2023). Migraine: advances in the pathogenesis and treatment. Neurol. Int. 15 (3), 1052–1105. 10.3390/neurolint15030067 37755358 PMC10535528

[B44] Rajendran A. Minhas A. S. Kazzi B. Varma B. Choi E. Thakkar A. (2023). Sex-specific differences in cardiovascular risk factors and implications for cardiovascular disease prevention in women. Atherosclerosis 384, 117269. 10.1016/j.atherosclerosis.2023.117269 37752027 PMC10841060

[B45] Russo A. F. Hay D. L. (2023). CGRP physiology, pharmacology, and therapeutic targets: migraine and beyond. Physiol. Rev. 103 (2), 1565–1644. 10.1152/physrev.00059.2021 36454715 PMC9988538

[B46] Sherwood A. Hill L. K. Blumenthal J. A. Johnson K. S. Hinderliter A. L. (2017). Race and sex differences in cardiovascular α-adrenergic and β-adrenergic receptor responsiveness in men and women with high blood pressure. J. Hypertens. 35 (5), 975–981. 10.1097/HJH.0000000000001266 28306633 PMC5785915

[B47] Spies M. Handschuh P. A. Lanzenberger R. Kranz G. S. (2020). Sex and the serotonergic underpinnings of depression and migraine. Handb. Clin. Neurol. 175, 117–140. 10.1016/B978-0-444-64123-6.00009-6 33008520

[B48] Steiner T. J. Stovner L. J. Jensen R. Uluduz D. Katsarava Z. Lifting The Burden: the Global Campaign against Headache (2020). Migraine remains second among the world's causes of disability, and first among young women: findings from GBD2019. J. Headache Pain 21 (1), 137. 10.1186/s10194-020-01208-0 33267788 PMC7708887

[B49] Terol-Úbeda A. C. Fernández-González J. F. Morán A. García-Domingo M. García-Pedraza J. Á. (2025a). Angiotensin II and EDH pathways underlie the vascular sympatho-modulation by 5-HT in female rats. Int. J. Mol. Sci. 26 (19), 9614. 10.3390/ijms26199614 41096879 PMC12524661

[B50] Terol-Úbeda A. C. Fernández-González J. F. Roldán-Hernández C. A. Martín M. L. Morán A. García-Domingo M. (2025b). Sex influence on serotonergic modulation of the vascular noradrenergic drive in rats. Br. J. Pharmacol. 182 (4), 1025–1037. 10.1111/bph.17380 39489611

[B51] Thakore P. Brain S. D. (2017). The role of perivascular adipose tissue-derived sensory nerves in influencing vascular regulation. Cardiovasc. Res. 113 (8), 847–848. 10.1093/cvr/cvx099 28863438

[B52] Vervoort D. Wang R. Li G. Filbey L. Maduka O. Brewer L. C. (2024). Addressing the global burden of cardiovascular disease in women: JACC state-of-the-art review. J. Am. Coll. Cardiol. 83 (25), 2690–2707. 10.1016/j.jacc.2024.04.028 38897679

[B53] Villalón C. M. Albarrán-Juárez J. A. Lozano-Cuenca J. Pertz H. H. Görnemann T. Centurión D. (2008). Pharmacological profile of the clonidine-induced inhibition of vasodepressor sensory outflow in pithed rats: correlation with alpha(2A/2C)-adrenoceptors. Br. J. Pharmacol. 154 (1), 51–59. 10.1038/bjp.2008.49 18297098 PMC2438980

[B54] Viticchi G. Falsetti L. Silvestrini M. Bartolini M. (2022). Ditans: a new prospective for the therapy of migraine attack? Neurol. Sci. 43 (9), 5709–5716. 10.1007/s10072-022-06260-z 35816257

[B55] Zhang Y. Wang N. Zhang L. Zhuang Y. Xin Q. Gu X. (2025). Serotonin (5-Hydroxytryptamine): metabolism, signaling, biological functions. Dis. Emerg. Ther. Oppor. MedComm. 6 (9), e70383. 10.1002/mco2.70383 PMC12421070 40937192

