# Correction: Sex-dependent modulation of CGRPergic neurovascular activity by 5−CT in rats

**DOI:** 10.3389/fphar.2026.1909543

**Published:** 2026-07-10

**Authors:** Anaïs Clara Terol-Úbeda, Asunción Morán, Mónica García-Domingo, Jose Ángel García-Pedraza

**Affiliations:** 1 Laboratorio de Farmacología, Departamento de Fisiología y Farmacología, Facultad de Farmacia,Universidad de Salamanca, Salamanca, Spain; 2 Instituto de Investigación Biomédica de Salamanca (IBSAL), Salamanca, Spain

**Keywords:** 5−CT, 5-HT_1F_ receptor, 5-HT_7_ receptor, CGRP, migraine, sex differences, vasodepressor sensory outflow

A correction has been made to **Materials and Methods**, *Animal preparation and study design*, Paragraph 2. This sentence previously stated:

“Then, rats were artificially ventilated with room air (50 strokes/min, 100 mL air/100 g) and catheters were placed in (i) jugular and femoral veins, for the continuous perfusion of agonists (methoxamine followed by 5-HT receptor agonists) and i.v. administration of 5-HT receptors antagonists; and (ii) the left carotid artery, coupled to a pressure transducer and connected to an e-corder 410 amplifier, for recording mean BP (MBP; mmHg) and heart rate (HR; beats/min, bpm) using Chart™ (v5.5.11; eDAQ) and LabChart™ (v7.2; ADInstruments) software.”

The corrected sentence appears below:

“Then, rats were artificially ventilated with room air (50 strokes/min, 1 mL air/100 g) and catheters were placed in (i) jugular and femoral veins, for the continuous perfusion of agonists (methoxamine followed by 5-HT receptor agonists) and i.v. administration of 5-HT receptors antagonists; and (ii) the left carotid artery, coupled to a pressure transducer and connected to an e-corder 410 amplifier, for recording mean BP (MBP; mmHg) and heart rate (HR; beats/min, bpm) using Chart™ (v5.5.11; eDAQ) and LabChart™ (v7.2; ADInstruments) software.”

The authors apologize for this error and state that this does not change the scientific conclusions of the article in any way.

The original article has been updated.

